# Molecular Interventions towards Multiple Sclerosis Treatment

**DOI:** 10.3390/brainsci10050299

**Published:** 2020-05-15

**Authors:** Athanasios Metaxakis, Dionysia Petratou, Nektarios Tavernarakis

**Affiliations:** 1 Institute of Molecular Biology and Biotechnology, Foundation for Research and Technology Hellas, Nikolaou Plastira 100, 70013 Heraklion, Greece; thanos_metaxakis@imbb.forth.gr (A.M.); dipetratou@imbb.forth.gr (D.P.); 2 Department of Basic Sciences, Faculty of Medicine, University of Crete, 71110 Heraklion, Greece

**Keywords:** B cell receptor, delivery methods, immunotherapy, monoclonal antibodies, multiple sclerosis, T cell receptor, tolerance, vaccine

## Abstract

Multiple sclerosis (MS) is an autoimmune life-threatening disease, afflicting millions of people worldwide. Although the disease is non-curable, considerable therapeutic advances have been achieved through molecular immunotherapeutic approaches, such as peptides vaccination, administration of monoclonal antibodies, and immunogenic copolymers. The main aims of these therapeutic strategies are to shift the MS-related autoimmune response towards a non-inflammatory T helper 2 (Th2) cells response, inactivate or ameliorate cytotoxic autoreactive T cells, induce secretion of anti-inflammatory cytokines, and inhibit recruitment of autoreactive lymphocytes to the central nervous system (CNS). These approaches can efficiently treat autoimmune encephalomyelitis (EAE), an essential system to study MS in animals, but they can only partially inhibit disease progress in humans. Nevertheless, modern immunotherapeutic techniques remain the most promising tools for the development of safe MS treatments, specifically targeting the cellular factors that trigger the initiation of the disease.

## 1. Introduction

Multiple sclerosis (MS) is the commonest inflammatory autoimmune disorder of the central nervous system (CNS), progressively leading to demyelination, neurodegeneration, and neuronal disability [1,2,3]. MS globally affects more than 2.5 million people and it often afflicts young people, mainly women [4,5]. Despite the availability of a large arsenal of putative therapeutic approaches, numerous studies in animal model systems, and clinical trials, MS is still non-curable. As a result, the average life expectancy of MS patients is shorter by 5 to 10 years [6].

Inflammatory lesions at the CNS, generated by autoreactive lymphocytes, are suggested to underlie the pathophysiology of the disease, which results in neuronal demyelination and damage. Genetic and environmental factors influence MS susceptibility: Family history, single nucleotide polymorphisms, Epstein–Barr virus (EBV) infection, smoking, obesity, and vitamin D shortage are associated with MS development [7,8,9,10,11]. Patients experience relapsing-remitting phases of the disease, which are followed, even years later, by a progressive phase, accompanied by neurodegeneration [12,13]. MS symptomatology largely varies among patients, including sensory disturbances, cognitive defects, loss of vision, weakness, bladder dysfunction and neurological disability among others [14,15].

Therapeutic strategies against MS have been mainly relied on immune function suppressors, such as glucocorticoids, methotrexate, and antihistamines, which non-specifically reduce immune activity. These strategies have been enforced in recent years by the usage of antibodies against proinflammatory mediators [16]. However, this approach has severe side effects and dangers for patients, since the general inhibition of immune responses risks the development of infections and tumors. Hence, modern therapeutic approaches must aim at disease-modifying interventions that will counteract specifically the excessive immune response against self-antigens. Administration of self-antigens, an intervention that has been successfully applied in other autoimmune diseases and has been shown to eliminate the autoimmune response, is a widely accepted methodology to achieve this [17]. A major drawback of this technique is the poor targeting of CNS by the exogenously supplied antigens, for their inability to cross the brain–blood barrier and increased degradation. As such, the improvement of delivery methods used to protect and adequately transfer self-antigens to the inflammation sites has been an intriguing research field [18]. Nevertheless, a prerequisite for the success of this approach is that the epitope of the self-antigen is known. This is not true in the case of MS yet, although proteins within the myelin sheath have been suggested to be promising candidates [19,20]. Consequently, much research effort must be invested before modern immunomodulatory approaches can assure the cure of MS.

Recent experimental studies and clinical trials show that modern immunotherapeutic techniques have the potential to treat MS with less or no side effects in the future. Extensive work in mammalian model organisms has given insights into the mechanisms of the disease development and efficiency of several drugs in animals and humans. Indeed, novel drugs, such as Glatiramer acetate (Copaxone), a random sequence of four synthetic polypeptides with similar immunogenic properties to myelin protein, are currently being used against MS with very promising results [21]. In this review, we discuss antigen-specific and cell-specific immunotherapeutic approaches, applications of monoclonal antibodies against MS, anti-inflammatory strategies, peptide delivery methodologies and biological mechanisms that can serve as targets for the development of adjunctive MS treatments.

## 2. Immunotherapeutic Approaches

### 2.1. Antigen-Specific Immunotherapy (ASI)

Antigen-specific immunotherapy (ASI) is a promising strategy to treat MS with the least possible side effects. It was firstly introduced several decades ago, when Leonard Noon suppressed conjunctival sensitivity to grass pollen through prophylactic inoculation with grass pollen extracts [22]. His work paved the way for the first clinical trial of allergen immunotherapy a few decades later [23,24]. Allergen immunotherapy is based on the prevention of immune over-reaction against an allergen when repetitive doses of the latest are supplied to the organism. Repeated exposure to increasing amounts of an allergen results in altered cytokine production and shifts the immune response from a T helper 2 (Th2) to a T helper 1 (Th1) response, and also in the activation of regulatory T cells (Tregs) that secrete interleukin (IL)-10 and transforming growth factor (TGF)-β [25].

Contrary to allergic responses, where Th2 immune responses prevail, in autoimmune diseases, the prevalent responses are Th1 and Th17 against self-antigens. ASI for MS aims to induce Tregs in order to promote autoantigen-specific tolerance. The elimination of pathogenic Th1 and Th17 cells or the inhibition of the autoantigen-specific T cells-induced immune response might be the treatment for MS. Through repeated exposure to antigens, both allergen immunotherapy and ASI aim to promote self-tolerance [26].

Inspired by the progress in allergen immunotherapy, researchers have aimed at treating MS through the administration of self-peptides, which are expected to mimic the immunogenicity of self-antigens. This technique is called ‘peptide vaccination’ and promises to eliminate the antigen-specific attack without diminishing the organism’s immune capacity against other threats. The most successful peptide vaccines applied so far are fractions of myelin proteins, such as myelin basic protein (MBP), myelin oligodendrocyte glycoprotein (MOG), and proteolipid protein (PLP) [27]. These antigens have been used to induce autoimmune encephalomyelitis (EAE) in mouse models, a widely accepted inflammatory model used to study MS. Several trials of myelin self-antigen peptide vaccines have cured EAE to a lesser or greater extent. Vaccination of an immunodominant epitope of myelin basic protein (MBP) (peptide 87–99), shown to be recognized and attacked by the T cell receptor (TCR), prevented and treated EAE, while it reduced tumor necrosis factor (TNF)-alpha and interferon (IFN)-gamma production, two determinant cytokines in the pathogenesis of EAE and MS [28]. More MBP peptides are shown to be immunogenic, and upon vaccination, they can mildly or strongly counteract EAE pathogenesis [29]. Myelin PLP (peptide 139–151) peptides can also prevent or treat EAE in animals [30,31]. A peptide from another myelin protein, the myelin oligodendrocyte glycoprotein (MOG) (peptide 35–55), can inhibit EAE development in mice [32,33], similarly to peptides derived from proteolipid protein (PLP) [34,35]. Hence, promising results from animal model systems have recommended peptide vaccination as a featured strategy to counteract MS.

In humans, two promising vaccination-based clinical trials with myelin peptides were safe and well tolerated by MS patients. Moreover, vaccination suppressed autoreactive responses and IFN-gamma production, while it significantly improved clinical disease measures. The activation of Langerhans cells and generation of IL-10-secreting cells are suggested to underlie these effects [36,37]. *Chataway et al.* showed that a mixture of peptides derived from MBP (peptide ATX-MS-1467) was safe and well tolerated by MS patients, while it improved radiographic activity in magnetic resonance imaging (MRI) [38]. *Crowe et al.* used a fragment of MBP (peptide 83–99) to induce immune responses and enhance anti-inflammatory cytokine secretion from T lymphocytes that cross-react with MBP [39]. Similarly, subcutaneous administration of a mixture of three MBP peptides (peptides 46-64, 124–139, and 147-170), termed Xemys, in MS patients was safe, while treatment decreased the cytokines monocyte chemoattractant protein-1, macrophage inflammatory protein-1β, and IL-7 and -2 levels, thus indicating reduced inflammation. However, clinical parameters were not significantly changed in patients [40]. In another scheme, researchers vaccinated MS patients with autologous peripheral blood mononuclear cells, chemically coupled with seven myelin peptides. Administration of antigen-coupled cells did not cause adverse effects, it was well tolerated and patients exhibited decreased antigen-specific T cell responses after treatment [41].

Contrary to the above, some studies show that peptide vaccination can have severe side effects and few clinical trials have not been completed for safety reasons. In two studies, MBP peptide 83–99 not only did not improve the disease state of MS [42], but even aggravated it, with few patients having exacerbations of MS [20]. Furthermore, administration of myelin epitopes has raised safety concerns of anaphylaxis [43,44,45]. In conclusion, specific attention should be paid to the adverse effects of peptides vaccination and future studies must identify the factors underlying the diversity of evoked responses in MS patients. Genomic profiling of MS patients that develop such effects can indicate factors that underlie the toxicity of this approach and indicate complementary treatments to reduce side effects. Moreover, trials with novel immunogenic peptides and further experimentation on the timing and dosage of vaccination can improve the efficiency and reduce the adverse effects of peptides vaccination.

Another immunotherapy technique that has been applied to induce self-tolerance in MS patients is the administration of genetically engineered DNA that encodes human MBP protein (BHT-3009). Experiments with animals clearly highlighted the potential of DNA vaccination as a safe and efficient technique at inducing regulatory T cells and EAE inhibition in animals. Its application in MS patients was safe and well tolerated, thus offering an alternative to peptide vaccination in terms of safety. Moreover, it decreased the proliferation of IFN-gamma-producing myelin-reactive T cells, the number of myelin-specific autoantibodies in the cerebrospinal fluid, and MRI-measured disease activity, while it increased the antigen-specific tolerance to myelin-specific B and T cells [46,47,48,49]. Nevertheless, no significant clinical improvements in the disease development were observed in these trials.

### 2.2. Cell-specific Immunotherapy

T cell vaccination is another immunotherapeutic approach, which is aimed at reducing or inactivating pathogenic T cells that maintain an autoimmune attack on myelin in MS. T cells’ reaction is believed to be the initial step that drives the pathogenesis of MS [50]. In this technique, autologous myelin-reactive T cells are isolated and inactivated prior to their administration to MS patients. Initial trials clearly showed safety and encouraging effects from T cell vaccination [51]. In a matched trial, MS patients were vaccinated with irradiated MBP-reactive T cells. Vaccinated patients with relapsing-remitting disease phases experienced a remarkable decrease in disease exacerbations and a five-fold lower increase in brain lesion size, compared to controls [52]. In three cases, however, T cell vaccine aggravated brain lesions and worsened relapses, a condition accompanied by reactivation of circulating MBP-reactive T cells. *Zhang et al.* showed that inhibition of MBP-reactive T cells was correlated with a 40% reduction in the rate of disease relapses, while brain lesion activity in vaccinated patients was stabilized [53]. This trial revealed that repetitive T cell vaccinations are needed to hamper the reappearance of myelin-reactive T cell clones.

Alternative T cell vaccination schemes use mixtures of inactivated autoreactive T cells, selected with more than one myelin peptides. In one trial, T cells activated with synthetic MBP and MOG peptides were administrated in MS patients, with no adverse effects being reported. Patients exhibited stabilized neurological symptoms and vaccination reduced active brain lesions both in number and size [54,55]. Tcelna (formerly known as Tovaxin) is a T cell vaccine containing T cell populations selected with peptides derived from MBP, PLP, and MOG. In a double-blind trial involving a restricted number of MS patients, vaccination did not cause adverse effects and showed mild clinical efficacy [56]. More studies are required to properly evaluate the potency of Tcelna to treat MS.

Another suggested methodology to inhibit the autoimmune response in MS is via the elimination of dendritic cells, which play a major role in inflammation induction. Dendritic cells are the most efficient antigen-presenting cells (APCs) of the immune system and they have a particular role in the stimulation of naïve T cells. They regulate T cell differentiation and priming, secrete proinflammatory cytokines, orchestrate the immune response against self-antigens, and initiate chronic inflammation and loss of tolerance [57]. Dendritic cells respond occasionally to a specific antigen, in a manner dependent on the tissue environment. Tolerance-inducing (Tolerogenic) dendritic cells are dendritic cells with immunosuppressive properties, elicited by the induction of T cell anergy, T cell apoptosis, regulatory T cell activity, and production of anti-inflammatory cytokines [58]. In vitro treatment of monocyte-derived dendritic cells with vitamin D3 causes T cell hyporesponsiveness to myelin [19,59]. MOG 40–55 peptide-treated tolerogenic cells that were administrated in mice preventively or after EAE induction reduced incidence of the disease or improved its clinical features, respectively [60]. Several trials in humans show that the technique is safe in patients with other autoimmune diseases [19]. Recently, engineered dendritic cells, loaded with specific antigens, were used to induce tolerance in MS patients. Therapy was safe and well tolerated; it increased IL-10 levels and the number of regulatory T cells, indicating that antigen-specific tolerance can be, at least partially, induced with this approach [61].

### 2.3. Cell Receptor-Specific Immunotherapy

A similar approach to cell-specific immunotherapy is T cell receptor-specific immunotherapy. Here, fragments of the T cell receptor (TCR) from pathogenic T cell clones are used as peptide vaccines, in order to activate immune responses against TCR-expressing T cells. TCR is a protein complex that recognizes antigens bound to major histocompatibility complex (MHC) molecules. Different TCRs can be specific for the same antigen, while more than one antigen peptides can be recognized by the same TCR [62].

Vaccination of rats with a synthetic TCR V-region peptide conferred resistance to subsequent induction of EAE [63]. According to the study, T cells specific for the TCR peptide weakened the immune attack to the encephalitogenic epitope. Furthermore, *Offner et al.* showed that TCR vaccination can not only prevent EAE but also cure it. When a TCR-V beta 8-39-59 peptide was injected into rats with EAE, disease symptoms were alleviated and recovery from the disease was fast [64].

To test safety and immunogenicity of TCR vaccines in humans, *Bourdette et al.* intradermally injected MS patients with two synthetic TCR peptides (TCR peptides V beta 5.2, 39-59 and V beta 6.1, 39-59). Low doses of the TCR vaccine caused no side effects, restricted spectrum immunosuppression, generated TCR peptide-specific T cells, and reduced MBP-specific T cells [65]. In a subsequent trial, TCR vaccination enhanced TCR-reactive T cells, reduced the MBP response against MBP antigen, stabilized clinical features, and caused no adverse effects to MS patients [66]. In support, TCR-specific Th2 cells inhibit the MBP-specific Th1 response in vitro through the release of IL-10, and a triplicate TCR vaccine (BV5S2, BV6S5, and BV13S1 peptides) increases the numbers of circulating IL-10-secreting T cells, reactive to the TCR peptides, in MS patients [67].

Together with pathogenic T cells, autoreactive B cells are involved in MS induction. Hence, the B cell receptor (BCR) can be used as a vaccine as well. Single-cell sequencing and phage display libraries of B cells derived from MS patients have been performed to identify BCR structures involved in MS autoimmunity [68,69,70]. *Gabibov et al.* showed that, antibodies induced against Epstein–Barr virus latent membrane protein 1 (LMP1) potentially react with MBP. This suggests that natural molecular reactivity might underlie MS induction and raises questions about the causal link between virus infection and MS development. Recently, antibody engineering techniques have allowed for the targeting of BCR with toxins, resulting in the cell death of pathogenic B cells [29,71,72]. This makes BCR-specific immunotherapy an alternative, although still at a preliminary state, approach to treat MS.

### 2.4. Monoclonal Antibodies (MABs)

The usage of monoclonal antibodies is another encouraging molecular therapy against MS, for their high specificity and high efficacy. Several ones have been approved for MS treatment [73,74]. Natalizumab, an adhesion molecule inhibitor, was the first MAB to be approved in 2004 [75]. It is a recombinant humanized MAB that binds integrin α-4 on the surface of activated inflammatory lymphocytes and monocytes. This inhibits the interaction of integrin a-4 with vascular cell adhesion molecule-1 (VCAM-1) on endothelial cells and consequently circulation into the CNS. Clinical trials show that it is safe, well tolerated, and efficient, since it reduces the risk of sustained progression of disability and MS relapses [76]. Ocrelizumab and Rituximab are MABs that target CD20 protein on B lymphocytes. They have been shown to reduce the rates of disease activity and disease progression [77,78]. Ofatumumab also binds on CD20, albeit at a different epitope, and its administration in MS patients reduces new MRI-detected lesions by 99% [79]. Another MAB, Opicinumab, has been designed to repair and enhance re-myelination of lesions in MS patients. Opicinumab is a fully humanized MAB that targets and inactivates leucine rich repeat and immunoglobin-like domain-containing protein 1 (LINGO-1), a transmembrane signaling protein that inhibits the differentiation of oligodendrocytes and myelination. Hence, it is potentially a promising tool to induce re-myelination in MS patients and alleviate disease symptoms. It has been tested in mice and in humans, where it increases myelination and re-myelination in MS patients [80,81]. Alemtuzumab is a humanized monoclonal antibody, approved in several countries for the treatment of relapsing-remitting MS. It targets CD52 antigen on lymphocytes, resulting in their depletion [82]. Hence, monoclonal antibodies are very promising tools for MS therapy for their safety, specificity, and efficacy but also for the various cellular procedures they can target to reduce autoimmunity and its clinical consequences.

### 2.5. HLA Antagonistic Co-polymers

Synthetic materials (copolymers) can mimic the immunogenic properties of endogenous proteins and compete with them for binding to HLA class II molecules. Glatiramer acetate (Copaxone or GA) is a random polymer of four amino acids (L-alanine, L-glutamic acid, L-lysine, and L-tyrosine) that effectively treats experimental encephalomyelitis and reduces relapses in MS patients [83,84,85]. GA is suggested to specifically inhibit the production of myelin-reactive antibodies, by directly acting on APCs. This modifies them into non-inflammatory type II cells. APCs-mediated presentation of GA to CD8+ and CD4+ T cells results in the generation of CD4+ regulatory T cells and immune response deviation towards Th2 responses [86,87]. A second generation of polymers has been synthesized with stronger binding activities on HLA molecules compared to GA. They have been successfully used to suppress EAE in mice [88]. In transgenic mice with human HLA-DR-TCR, poly(VWAK)n copolymers are shown to induce T cells’ anergy, while poly(FYAK)n copolymers induce Th2 cells that secrete anti-inflammatory cytokines [29]. Hence, they can serve as alternative tools for shifting the immune response towards Th2 activation in MS patients.

## 3. Delivery Methods of Immunotherapeutic Factors

A key point for the successful implementation of immunotherapy treatment is the efficacy of the delivery methodology. Oral, skin, parenteral, intramuscular, intravenous, and intra-peritoneal routes are mainly used with various delivery vehicles. These vehicles must enhance the tolerance of immunomodulatory molecules against the harsh intra-organismal environment and advance their efficacy to overcome the brain–blood barrier. Synthetic polymers, such as poly lactide-co-glycolide (PLGA), polyethylene glycol (PEG), and polymethylmethacrylate (PMMA), are easily synthesized and modified, capable of transferring sufficient amounts of immunotherapeutic molecules and facilitating their gradual release [18]. Permeability is decreased when electrically charged nanoparticles are used, such as orally administrated polyethylene imine-based nanoparticles and thiol-modified Eudragit polymers (polymethacrylates) [89,90]. Transgenic plant delivery is another technique that takes advantage of the protective effect of the plant cell wall, especially for delivery through the gastrointestinal tract [91,92]. Nanoemulsions, small colloidal particles, provide a high encapsulation efficiency [93], while phosphatidylserine-liposomes have been efficiently used to reduce EAE severity in mice [94]. Much attention has been paid to lipid-based nanocarriers, such as nanoemulsions, nanoliposomes, solid lipid nanoparticles (SLNs), and nanostructured lipid carriers (NLCs), which are suggested to be efficient for brain targeting. NLCs have been reported to be very safe and stable, with a high encapsulation efficiency [95,96]. A major challenge in the field of immunotherapy treatment is the improvement of delivery methods so that immunotherapeutic molecules can be transferred more efficiently through the brain–blood barrier. This will improve the therapeutic efficiency, reduce side effects, and decrease the number of administration procedures. More selective delivery to the CNS can be achieved through the covalent tethering of delivery molecules with ligands capable of overcoming the brain–blood barrier, the use of fusion antibodies that target specific lymphocytes, and of liposomes that intrinsically tend to reach inflammation sites.

Therapeutic treatments for MS target lymphocyte subpopulations, specific for autoreactive response towards the myelin sheath. Tolerogenic DCs, myelin peptide and DNA vaccines, TCR peptides and GA lead to the activation of Th2 cells, through Tregs. Subsequent release of IL-10 leads to the inhibition of Th1 cells. DMF acts on HCAR2, found on dendritic cells, to induce Th2 cells. Toxins targeting BCRs lead to the elimination of pathogenic B cells. Fingolimod blocks the circulation of mature lymphocytes through S1PR, and Teriflunomide and Mitoxantrone inhibit T and B cell proliferation. Anti-CD 20 and anti-CD 52 antibodies deplete CD 20+ and CD 52+ lymphocytes. Tolerogenic TCs block MBP-reactive T and B cells. Natalizumab binds to α 4 β 1 integrin on activated T and B cells and prevent their interaction with VCAM-1. Opicinumab promotes the differentiation of oligodendrocyte precursor cells by inactivating LINGO-1. Abbreviations: Antigen Presenting Cell (APC), Blood–Brain Barrier (BBB), B Cell Receptor (BCR), cluster of differentiation 52/20 (CD52/20), Dendritic Cells (DCs), DMF (Dimethyl Fumarate), Glatiramer Acetate (GA), hydroxycarboxylic acid receptor 2 (HCAR2), Interferon (IFN), Interleukin (IL), Immunoglobin-like domain-containing protein 1 (LINGO-1), MBP (Myelin Binding Protein), MMF (Monomethyl Fumarate), Multiple Sclerosis (MS), Sphingosine-1-phosphate receptor (S1PR), TCR (T cell Receptor), T helper 2 cell (Th2), T helper 1 cell (Th1), T Cell Receptor (TCR), T regulatory cells (Tregs), TCs (T cells), vascular cell adhesion molecule-1 (VCAM-1). (Table 1).

## 4. Conclusions

**Figure 1:**
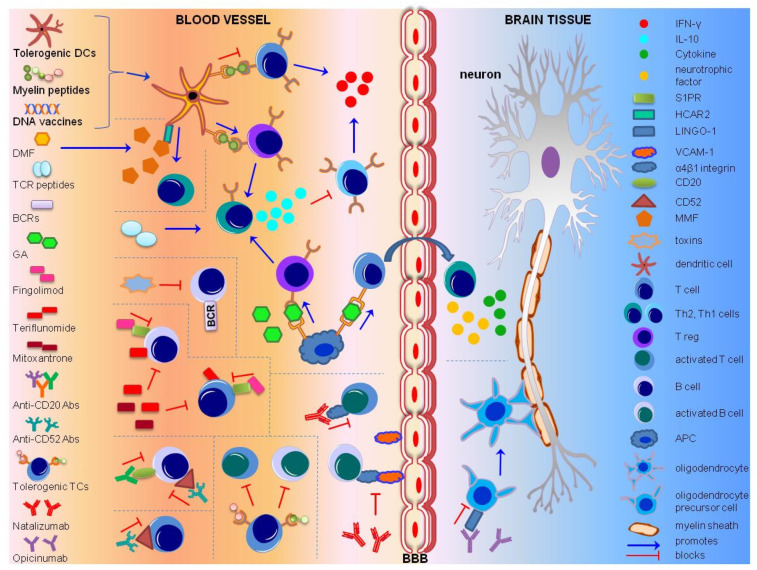
Mechanism of action of immunomodulatory treatments for multiple sclerosis.

Researchers in the field of MS treatment have been trying to cure the disease via the elimination of CNS inflammation, elicited by the MS-related autoimmune response. Different applied strategies include the deviation of the immune response towards non-inflammatory Th2 activation, inactivation or amelioration of cytotoxic autoreactive T cells, induction of anti-inflammatory cytokines’ secretion, inhibition of inflammatory cytokines, blockage of autoreactive-lymphocytes’ recruitment to the CNS, and enhancement of myelination mechanisms (Figure 1). Several drugs have been tested so far in clinical trials, some of which can reduce relapses and symptoms in MS patients (Table 1), thus significantly improving their quality of life. However, none of them can cure MS. Despite the success of allergen immunotherapy in treating allergies, ASI has not displayed great achievements so far as a putative MS treatment. Reasons underlying this might be the difficulty in the identification of the self-antigens that trigger autoimmunity, the inability of regulatory T cells to suppress cytokine production under inflammatory conditions, the different immune players participating in allergies compared to MS (e.g., IgE antibodies, Th2 responses), and also the route, dosage, and timing used for ASI treatments [128]. Nevertheless, more than 10 drugs are currently being used against the secondary progressive form of MS, characterized by the relapsing-remitting phases, significantly reducing the frequency of relapses and disease symptoms [14]. These drugs are either immunosuppressants (such as Natalizumab, Ocrelizumab, Fingolimod, Alemtuzumab) or immunomodulatory (such as Interferon beta, GA, Teriflunomide, Mitoxantrone, Dimethylfumarate). Fingolimod reduces the number of circulating mature lymphocytes [129], Teriflunomide and Mitoxantrone are inhibitors of lymphocytes proliferation and the secretion of cytokines [130,131], while Dimethylfumarate (DMF), used for psoriasis treatment, shifts the Th1 and Th17 immune responses to Th2 [132]. However, these drugs do not cure the primary progressive form of MS, they must be repetitively supplied to the MS patients, and they can have adverse effects. As such, more selective and efficient drugs are required to assure safe treatment of MS in the future.

Basic research on the mechanisms that underlie MS can reveal novel targets for monoclonal antibodies, identify the specific self-antigens that trigger autoimmunity, and characterize the types of lymphocytes that participate in the inflammatory reaction, so that antigen and cell-specific immunotherapies expand and become more precise. In addition, the identification of novel carriers or ligands that, upon conjugation, will lead these immunotherapeutic molecules to the CNS inflammatory sites can improve the efficiency of treatments. It is also important to clarify the role of Epstein–Barr virus infection on MS development and their possible association, which might give further insights into the disease etiology and treatment. Improved delivery of therapeutic molecules is another challenge of research in MS, which can be achieved through the generation of fusions between the therapeutic molecules and peptide leaders that will efficiently guide them to the inflammation sites in the brain [133]. Recently, a fusion protein of an NOD-like receptor family member X1 (NLRX1) and blood–brain barrier-permeable peptide dNP2 treated experimental autoimmune encephalomyelitis in mice [134] and a peptide that selectively recognizes the CNS was used for targeted drug delivery to the CNS in mice [135]. Genome-wide DNA sequencing analysis of MS patients is another approach that can advance our knowledge on the disease etiology and on MS patients’ responses to medical treatments; it can reveal genes that make people more susceptible to MS and identify the reasons why specific drug treatments have adverse effects in some patients. In this case, the proper therapy could be administrated to patients that have certain genetic profiles, so that adverse effects of MS therapy could be minimized. Furthermore, drugs that enhance myelination, such as metformin [136], growth factors shown to regulate inflammation [137], and hormones known to affect autoimmunity [138] can offer new perspectives into the development of novel complementary treatments of MS in the future.

## Figures and Tables

**Table 1:** Overview of medical treatments for multiple sclerosis.

Treatment	Mode of Action	MS Type	Study Format (Number of Participants)	Clinical Outcomes	Adverse Effects	Administration Route	References
**Interferons**							
Interferon-β1a *	reduces immature-transitional B cell subset/plasmablasts ratio, increases CD27^-^ and CD27^+^IgM^+^ memory B cell subsets, enhances Tregs	RRMS	case-control study/multicenter, open-label, prospective clinical trial, phase 4 (96)	reduction in relapse rates, reduction in MRI measurement of disease, well tolerated	flu-like symptoms, asthenia, fever, malaise, fatigue, local pain at the injection site	intramuscular injection	[97,98]
Interferon-β1b *	reduces neuron inflammation	RRMS	multicenter, randomized, double-blind, placebo-controlled trial (372)	reduced ARR, and MRI lesions	lymphopenia, skin reactions to injection, flu-like symptoms, fever, chills, myalgia, sweating, malaise	subcutaneous injection	[99,100]
**Peptides**							
**Peptide loaded cells**							
Myelin peptides (MOG1-20, MOG35-55, MBP13-32, MBP83-99, MBP111-129, MBP146-170, PLP139-154)	myelin peptide coupled autologous peripheral blood mononuclear cells, slightly increase T regulatory cells	RRMS SPMS	open-label, single-center, dose-escalation study, phase 1 trial (9)	safe and well tolerated	metallic flavor during infusion and IARs (diarrhea, headache, diverticulitis of sigma, neck pain, vision disturbance, dysesthesia, cold, gastric pain)	infusion	[41]
**Peptide vaccines**							
NBI-5788	altered MBP83-99 peptide, induces Th2-like cells APL-reactive	PPMS SPMS RRMS	multicenter phase 1 trial (11)	induced NBI-5788 responsive T cells, no clinical exacerbations	-	subcutaneous infusion	[39]
Xemys	mannosylated liposomes encapsulating MBP peptides, increases TNF-α, cytokine’s levels normalization	RRMS SPMS	phase 1 trial (18)/phase 1, open-label, dose-escalating, proof-of-concept study (20)	increased TNF-α serum levels, safe and well tolerated	injection site reaction, rhinitis, general weakness	subcutaneous infusion	[40,101]
peptides MBP85-99, MOG35-55, and PLP139-155	induce T regs producing IL-10, reduce IFN-γ and TGF-β	RRMS	double-blind, placebo-controlled cohort study (30)	reduced GdE lesions and ARR	local skin reaction (redness, itching), upper respiratory tract infection, lacrimation	transdermally, with skin patch	[36,37]
ATX-MS-1467	peptide mixture of MBP derived epitopes, induces MBP tolerance and IL-10 secreting T regs	RMS	multicenter, phase 1b (43), phase 2a, multicenter, single-arm trial (37)	reduced GdE lesions	erythema, induration, pain, pruritus, hemorrhage, alopecia, diarrhea	intradermal/ subcutaneous injection	[38,102]
**DNA vaccine**							
BHT-3009	decreases T cells	RRMS	randomized, multicenter, double-blind, placebo-controlled dose escalation, phase 1/2 trial (30)/randomized, placebo-controlled, phase 2 trial (289)	reduced GdE lesions, reduced myelin-specific autoantibodies, safe and well tolerated	infections, musculoskeletal, urinary, gastrointestinal psychiatric, respiratory effects (IARs)	intramuscular injections	[47,48]
**TCR vaccines**							
TCR V beta 5.2, 39-59 and V beta 6.1, 39-59	induce T regs	PMS	dose escalation study (11)	induced T cell immunity to synthetic peptides, safe	skin hypersensitivity reaction to the injection, no side effects or broad immunosuppression	intradermal injection	[65,103]
vβ5.2-38-58	induce Th2 cells and inhibits MBP-specific Th1 cells	PMS	double-blind (23)	induced T cell immunity to synthetic peptides, attenuated disease progression	no side effects or broad immunosuppression	intradermal injection	[66]
BV5S2, BV6S5 and BV13S1	induce IL-10 secreting T cells	RRM PMS	single-arm, open-label study (23)	induced T cell immunity to synthetic peptides, stabilized disease, improved FoxP3 expression, safe	no side effects	intramuscular injection	[67]
**Monoclonal antibodies**							
Natalizumab *	anti-a4-integrin Ab, prevents leukocytes crossing BBB	early RRMS	controlled, non-randomized trial (34)/multicenter, observational, open-label, single-arm, phase 4 study (222)	reduced relapse rates, MRI lesions and progression of disability, improvement in information processing speed, NEDA, SDMT and MSIS-29 physical, psychological and quality-of-life	suicide attempt, acute kidney injury, anaphylactic reactions, bronchial obstruction, clostridium difficile colitis, conversion disorder, hydronephrosis, hyperkaliemia, hypotension, ileus, melanoma recurrent, migraine	intravenous infusion	[104,105]
		SPMS	randomized, double-blind, placebo-controlled, phase 3 trial (889), open-label extension (291)	reduced progression of disability, improved ARR and MRI measurements, well tolerated	urinary tract infection, nasopharyngitis, fall, MS relapse, headache, fatigue, upper respiratory tract infection, back pain, arthralgia, pain in hands and feet, muscular weakness (IARs)	intravenous infusion	[106]
Opicinumab	anti-LINGO-1 Ab, allows oligodendricy maturation	RRMS SPMS	double-blind, dose-ranging, proof-of-concept, phase 2b study (418)/phase 1, randomized, multiple ascending dose study	primary endpoint was not met, inverted U-shaped dose-response	unaffected immune function	intravenous infusion	[81,107]
Alemtuzumab*	anti-CD52 IgG Ab, depletes circulating T and B lymphocytes	RRMS	rater-masked, randomized, controlled phase 3 trial (667)	reduced ARR, stabilized disability levels, improved clinical and MRI outcomes, reduced brain volume loss	infections, thyroid-associated adverse events, thrombocytopenia IARs (headache, pyrexia, rash, bradycardia, insomnia, erythema, nausea, Urticaria, pruritus, abdominal pain, fatigue, dyspnea, flushing)	intravenous infusion	[108]
Ofatumumab	anti-CD20, cytotoxic to B lymphocytes	RRMS	randomized, double-blind, placebo-controlled, phase 2 study (36)/randomized, double-blind, phase 2b study (232)	decreased new MRI lesions, safe	rash, erythema, upper respiratory tract infection, viral infection, throat irritation, headache, fatigue, back pain, flushing, injection related reactions	subcutaneous injection	[79,109]
Rituximab	selective depletion of CD20+ B lymphocytes	PMS	single-center, open-label trial (8)/retrospective, uncontrolled, observational, multicenter study (822)	reduced peripheral B cells, CSF B cells and CXCL-13 levels, increased BAFF levels/ lower EDSS score, delayed CDP	IARs (lower extremity paresthesia), lower extremity spasticity or weakness, fatigue, fever, rigors/ infections (respiratory, intestinal), disorders (cardiac, respiratory, neuronal, immune) and IARs (malaise, headache, chills, nausea)	intrathecal infusion	[110,111]
		RRMS	blind, single-center, phase 2 trial (30)	reduced relapses and GdE lesions	IARs (fever, chills, flushing, itching of body or throat, and/or diarrhea, shortness of breath), urinary tract infections, thigh pain, upper respiratory tract infection, bronchitis, hand tendonitis, dizziness	intravenous infusion	[112]
		PPMS SPMS	multicenter, prospective, open-label phase 1b trial (23)/randomized, double-blind, placebo-controlled, multicenter, phase 2/3 trial (439)	well tolerated and feasible, reduced GdE lesions, delayed CDP	IARs (vertigo, nausea), infections, paresthesia, fall, nervous system disorders, fever, fatigue, meningitis/IARs (nausea, fatigue, chills, pyrexia, headache, dizziness, throat irritation, pharyngolaryngeal pain, pruritus, rash, flushing, hypotension), pneumonia, bronchitis	intravenous or intrathecal infusion	[113,114]
Ocrelizumab*	anti-CD20 Ab, depletes circulating CD20+ B cells	RMS PPMS	randomized, double-blind, active-controlled, phase 3 trials (1651), randomized, parallel-group, double-blind, placebo- controlled, phase 3 study (725)	reduced new and GdE lesions, improved ARR, disability progression, and MRI outputs	IARs (pruritus, rash, throat irritation, flushing, urticaria, oropharyngeal pain, headache, tachycardia, pyrexia, nausea, hypo-, hyper-tension, myalgia, dizziness, fatigue)	intravenous infusion	[115,116]
		PPMS	randomized, double-blind, placebo-controlled, phase 3 trial (732)	reduced risk of Upper Extremity disability progression, enhanced NEPAD, reduced brain volume loss	IARs (upper respiratory tract infections, oral herpes infections, pruritus, rash, throat irritation, flushing)	intravenous infusion	[117,118]
**HLA antagonistic co-polymers**							
Glatiramer acetate *	increases Tregs to suppress inflammatory response	RRMS	randomized, placebo-controlled, double-blind study (251), open-label (208)	reduced relapse rate, reduced GdE and new lesions	IARs (flushing, anxiety, dyspnea)	subcutaneous injection	[119]
**Sphingosine-1-phosphate receptor modulators**							
Fingolimod *	structural analogue of sphingosine, anti-inflammatory, impairs cytotoxic CD8 T cells function	RRMS	prospective observational study (60)	higher retention rate, increased satisfaction at MSQ, reduced dGM volume loss, ARR and EDSS	influenza-like illness, pain in extremity, headache, anxiety, depression, nasopharyngitis, hypoesthesia, arthralgia, dizziness, fatigue, rash, urinary tract infection, abdominal pain, hypertension, lymphopenia	oral	[120,121]
**Other inhibitors**							
Teriflunomide *	DHODH inhibitor, reduces proliferation of T- and B-cells	RMS	prospective, single-arm, open-label, phase 4 real-world study (1000)/randomized, double-blind, placebo-controlled, phase 3 trial (168)/multicenter, multinational, randomized, double-blind, parallel-group, placebo-controlled, phase 3 study (2251)	well tolerated, improved MRI outcomes, reduced ARR and CDW, improved TSQM scores, stabilized disability measures, improved cognition and quality of life measures	neutropenia, hair thinning, diarrhea, nausea, headache, urinary tract infection, increased alanine aminotransferase, nasopharyngitis, fatigue, paresthesia	oral	[122,123,124]
**T cell vaccination**							
MBP-reactive T cells	deplete circulating MBP-reactive T cells.	RRMSSPMS	pilot, controlled (8)/preliminary open label study (54)	safe and well tolerated, improved MRI outcome, reduced relapse rates	no adverse effects, skin infection	subcutaneous injection	[52,53]
MBB-, MOG-reactive T cells	deplete circulating MBP-, MOG-reactive T cells.	RRMS	20	improved MRI outcome	no adverse effects, skin infection	S subcutaneous injection	[55]
MBP-, MOG-, PLP-reactive T cells/ Tovaxin	deplete circulating MBP-, MOG-, PLP-reactive T cells.	RRMSSPMS	open-label dose escalation study (16)/randomized, double-blind trial, phase 2 study (26)	well tolerated, reduced EDSS, ARR and 10 min walking time, stabilized MRI lesions, improved EDSS and MSIS-29	relapse of MS, pain in extremity, IARs (injection site pain, erythema, inflammation, pruritus), unrelated to TCV administration (anemia, intestinal obstruction, pneumonia, carpal tunnel syndrome, headache, respiratory distress, infections)	Subcutaneous injection	[54,56]
**Dendritic cell vaccination**							
peptide loaded cells	increase T regulatory cells and IL-10 levels	RRMSSPMSPPMS	open-label, single-center, multiple ascending-dose, phase 1b trial (12)	well tolerated, stabilized disease progress	headache, leg pain, cold, palpitations, influenza (and unrelated to TCV administration)	intravenous	[61]
**Esters**							
Dimethyl Fumarate *	fumaric acid ester, modulates CD4(+) cells, M2 monocytes and B-cells, induction of antioxidant response	RRMS	randomized, double-blind, placebo controlled, phase 3 trial (213)/open-label, observational, phase 4 study (1105)	decreased EDSS, GdE and new lesions, reduced ARR, improved treatment satisfaction and quality of life measures	flushing, nausea, abdominal pain, diarrhea, gastrointestinal events, nasopharyngitis, infections, cardiovascular, skin and hepatic events, pruritus, rash, headache, fall, lymphopenia, breast cancer, MS relapse	oral delayed release	[125,126]
**Other Immunomodulators**							
Mitoxantrone *	a synthetic anthracenedione, inhibits T-cell, B-cell and macrophage proliferation	SPMS RRMS PRMS	multicenter, prospective, open-label, observational, phase 4 study (509)	reduced GdE lesions and relapse rate, improved EDSS	congestive heart failure, leukemia, amenorrhea, decreased ejection fraction, urinary tract infection	intravenous infusion	[127]

Table 1. The main MS treatments are summarized. Some of them are approved while others are still under clinical trial. Their mode of action and outcomes of some indicative clinical trials are tabulated. With asterisk (*) are indicated the MS medications approved by the FDA. Abbreviations: Antibody (Ab), Altered Peptide Ligand (APL), Annualized Relapse Rate (ARR), B-cell Activating Factor (BAFF), Blood-Brain Barrier (BBB), Confirmed Disability Progression (CDP), Confirmed Disability Worsening (CDW), CerebroSpinal Fluid (CSF), C-X-C motif chemokinebinding Ligand-13 (CXCL-13), DiHydro-Orotate DeHydrogenase (DHODH), deep Gray Matter (dGM), Expanded Disability Status Scale (EDSS), Gadolinium-Enhanced (GdE), Infusion-Associated Reactions (IARs), InterFeroN (IFN), InterLeukin (IL), Leucine rich repeat and Immunoglobin-like domain-containing protein 1 (LINGO-1), Myelin Basic Protein (MBP), myelin oligodendrocyte glycoprotein (MOG), Modified Fatigue Impact Scale (MFIS), Mental Health Inventory (MHI), Medication Satisfaction Questionnaire (MSQ), Multiple Sclerosis (MS), No Evidence of Disease Activity (NEDA), No Evidence of Progression or active Disease (NEPAD), proteolipid protein (PLP), Primary Progressive Multiple Sclerosis (PPMS), Relapsing Multiple Sclerosis (RMS), Relapsing-Remitting Multiple Sclerosis (RRMS), Sphingosine-1-phosphate receptor (S1PR), Symbol Digit Modalities Test (SDMT), Secondary Progressive Multiple Sclerosis (SPMS), T-helper-2 cell (Th2), T Cell Receptor (TCR), Transforming Growth Factor beta (TGF-β), T regulatory cells (Tregs), Treatment Satisfaction Questionnaire for Medication Version 1.4 (TSQM 1.4).
